# Community pharmacists' management of self-limiting infections: a simulation study in Akwa Ibom State, South-South Nigeria

**DOI:** 10.4314/ahs.v21i2.12

**Published:** 2021-06

**Authors:** Richard Mary Akpan, Emmanuel Imo Udoh, Samuel Emediong Akpan, Chioma Cynthia Ozuluoha

**Affiliations:** Faculty of Pharmacy, University of Uyo, P.M.B. 1017, Uyo, Akwa Ibom State, Nigeria

**Keywords:** Antibiotics, pharmacists, common cold, acute diarrhoea, community pharmacy, patient simulation

## Abstract

**Background:**

Inappropriate use of antibiotics, especially for treatment of self-limiting infections remains one of the major drivers of antibiotic resistance (ABR). Community pharmacists can contribute to reducing ABR by ensuring antibiotics are dispensed only when necessary.

**Objective:**

To assess community pharmacists' management of self-limiting infections.

**Methods:**

A purposive sample of 75 pharmacies participated in the study. Each pharmacy was visited by an investigator and a trained simulated patient who mimicked symptoms of common cold and acute diarrhoea, respectively. Interactions between the simulated patient and pharmacist were recorded by the investigator in a data collection form after each visit. Descriptive statistical analysis was carried out. Ethics approval was obtained from the state Ministry of Health Research Ethics Committee.

**Results:**

For common cold, 68% (51/75) of pharmacists recommended an antibiotic. Azithromycin, amoxicillin/clavulanic acid, and sulphamethoxazole/trimethoprim (43%, 24%, 20%, respectively) were the most frequently dispensed agents. For acute diarrhoea, 72% (54/75) of pharmacists dispensed one antibiotic, while 15% dispensed more than one antibiotic. The most frequently dispensed agent was metronidazole (82%), which was dispensed in addition to amoxicillin or tetracycline among pharmacists who dispensed more than one agent. In both infection scenarios, advice on dispensed antibiotics was ofered in 73% and 87% of the interactions, respectively.

**Conclusion:**

This study shows high rate of inappropriate antibiotics dispensing among community pharmacists. There is need for improved awareness of antibiotic resistance through continuing education and training of community pharmacists. Furthermore, the inclusion of antibiotic resistance and stewardship in undergraduate pharmacy curriculum is needed.

## Introduction

Irrational use of antibiotics, especially for treatment of self-limiting viral infections such as, upper respiratory tract infections (URTIs) and acute diarrhoea (characterised by watery, non-bloody, non-mucoid stool with or without mild fever) contributes to the development of antibiotic resistance.^1^–^3^ In low- and middle- countries (LMICs) countries, antibiotic recommendations for viral URTIs ranges from about 40 to 75% and for acute diarrhoea, from about 20 to 55%.^4^ Community pharmacists are the most accessible healthcare professionals to the public for advice on common infections in LMICs.^5^ This is due to accessibility, reduced healthcare cost relative to private or public hospital visits, long hours of operation and provision services to clients with minimal waiting time, as well as patients' trust in expertise and knowledge of pharmacists.^6^–^9^ Community pharmacists therefore have a pivotal role in ensuring appropriate use of antibiotics.^10^ Although their knowledge of antibiotics can contribute to reducing antibiotic resistance in the community,^11^ available evidence shows that community pharmacists dispense and/or sell antibiotics for self-limiting viral infections.^12^–^18^ Factors which have contributed to this practice include poor national medicines regulations, lack of awareness of antibiotic resistance, commercial interest of pharmacists, consumer demand and failure of pharmacists to educate patients on the dangers of self-medication or irrational antibiotic use.^19^,^20^

The pharmaceutical sector (academic, hospital, industrial and community/retail) in Nigeria is regulated by the Pharmacists Council of Nigeria (PCN).^21^ While the PCN provides guidelines and procedures for registration and licensing of pharmaceutical premises, including community pharmacies, the community pharmacists control the operation and practice in their premises. Limited data exist on the community pharmacists' management of common viral infections in Nigeria. This study sought to assess community pharmacists' management of symptoms of common self-limiting infections in Akwa Ibom state, Nigeria.

## Methods

### Study setting

The study was carried out in registered community pharmacies spread across the 31 local councils of Akwa Ibom State. Akwa Ibom is costal state located in South-South, Nigeria with a population of about six million.^22^ The study was carried out between October and December 2019.

### Study design

This was a patient simulation study to determine community pharmacists' antibiotic dispensing practices for self-limiting infections. Patient simulation methodology has been shown to overcome the biases associated with direct observation, self-completion questionnaire and other study designs that involve self-report, as well as increase the validity of study findings.^12^,^13^,^17^,^18^,^23^–^25^

### Sampling technique and sample size

This study was an aspect of a state-wide survey to assess community pharmacists' knowledge and perceptions of antibiotic dispensing and antibiotic resistance. The list of all registered pharmacies and their addresses in the state for the year 2019 was obtained from the office of Pharmaceutical Society of Nigeria (PSN), Akwa Ibom State branch. As of August 2019, there were 166 registered pharmacies in the state. A purposive sample of 75 community pharmacies took part in patient simulation study.

### Data collection

#### Patient simulation

Two simulated patients (400 level undergraduate pharmacy students), who were sufficiently trained to undertake the task mimicked the symptoms of self-limiting viral infections following a one-day training session. The students, who were not part of the research team practiced details of the infection scenarios and roleplayed with members of the research team after the training session.

### Infection scenarios and data collection

We simulated common cold and acute diarrhoea, conditions which do not benefit from antibiotics, but antibiotics are often dispensed to both adults and children.^1^,^14^,^26^–^29^ The scenarios were developed based on previous studies^17^,^18^ and treatment guidelines.^2^,^30^–^32^ Each community pharmacy was visited twice by an investigator and a simulated patient, who mimicked symptom of common cold on the first occasion. After two weeks, the same investigator visited the same pharmacy with the second simulated patient, who mimicked symptoms of acute diarrhoea.

In each of the visit, the simulated patient walked into the pharmacy and requested to see the pharmacist. Complaints of common cold or acute diarrhoea were made and medication(s) requested to alleviate symptoms. The simulated patients did not provide any other information except the pharmacist asked. Details of infection scenarios are provided in Table 1.

**Table 1:** Details of infection scenarios

Scenario detail	Additional information (if the pharmacist asked)	What pharmacist should do
1. The patient is a 23-year old male who has had acute (watery and non-bloody) diarrhoea for the past 24 hours. He visits the toilet every 3–4 hours. The patient requests some medicines to alleviate his symptoms.	1. Vomited twice in the past 24 hours 2. No mucus or blood in stools. 3. Abdominal cramps. 4. No fever 5. No loss of appetite. 6. Has not tried any medicines 7. Has not seen a doctor. 8. Didn't eat outside home and no family member has similar symptoms.	Likely self-limiting viral gastroenteritis 1. Antibiotic not indicated; should not be dispensed/sold. 2. Advice to take oral rehydration solution (ORS). 3. Counsel on ORS preparation method 4. Advice on personal hygiene such as, hand washing with soap. 5. May offer loperamide/simethicone and zinc supplement to decrease the duration of symptoms. 6. Advice to see a doctor if diarrhoea persists or if there are signs of dehydration (excessive thirst, dry mouth, deep yellow urine or little or no urine, and severe weakness, dizziness).^2^,^32^
2. The patient is a 22-year old female. She complains of sneezing, chills, fever, throat irritation, cough, mild body ache, nasal congestion, mild headache, post-nasal drip (mucus dripping down the throat) and watery eyes. She has had the symptoms for the past 2 days. She needs some medicines to alleviate his symptoms	1. Treated malaria the previous week 2. Has not tried any medicines 3. Has not seen a doctor 4. Not pregnant; not breastfeeding	Common cold is generally caused by viruses, especially rhinovirus 1. Antibiotic should not be dispensed/sold; not effective both in children and adults. 2. Advice patient to rest 3. May offer analgesics (Acetaminophen or NSAID) 4. May offer antihistamine/decongestant combinations 5. Advice patient on hand hygiene.^30^,^31^

Interactions between the simulated patients and the pharmacists were recorded covertly. Antibiotics recommended by pharmacists were purchased, except expensive branded agents such as, Zithromax^R^ (Pfizer azithromycin) were dispensed and there was no generic substitute. The simulated patient will leave, with the pharmacist believing that the simulated patient will return to purchase the recommended antibiotic. At the end of each visit, the investigator completed a data collection form. Information captured in the form included degree of history taking (Who for, What symptoms, How long, Any medicine tried, other Medication taken, WWHAM),^18^,^33^ whether antibiotic was recommended/sold, details of antibiotic recommended, whether the pharmacists provided advice on dispensed/sold antibiotic and whether any other recommendations (such as, over-the-counter medicines or referral to doctor) were made. Data collection form is provided as Additional file 1.

### Data analysis

Data collected were coded and entered into Microsoft Excel version 10 (Microsoft, Redmond, USA); descriptive statistical analysis (frequencies and percentages) was carried out.

### Ethical considerations

Ethics approval for the study was obtained from the Akwa Ibom State Ministry of Health Research Ethics Committee (MH/PRS/99/Vol.IV/693), prior to commencement of data collection. In this study, simulation of infection was conducted covertly to overcome Hawthorne effect (change in behaviour by the subjects of a study due to their awareness of being observed) and to overcome biases associated with direct observation or self-completion questionnaire. This methodology is widely employed in pharmacy practice research.^17^,^18^,^23^–^25^ No personal information of the pharmacists or pharmacies was collected, only pharmacists' responses to the simulated cases were recorded.

## Results

### Common cold

Majority of the community pharmacists asked questions to confirm who had the ailment and the presenting symptoms (53/75, 70.7% and 60/75, 80%, respectively). Seventy-seven percent (58/75) of pharmacists did not ask the simulated patient what action(s) had been taken before presenting at the pharmacy. Details of the history questions for common cold are as presented in Table 2.

**Table 2:** WWHWAM questions for common cold

Questions	Yes, n (%)	No, n (%)
	N = 75	N = 75
“Who is the medication for?”	53/75 (71)	22/75 (29)
“What are the symptoms?”	60/75 (80)	15/75 (20)
“How long have you had the symptoms?”	36/75 (48)	39/75 (52)
“What action has already been taken?”	17/75 (23)	58/75 (77)
“Are you taking any other medicine?”	21/75 (28)	54/75 (72)
“Other medical and lifestyle history?”	31/75 (41)	44/75 (59)

**Figure 1:**
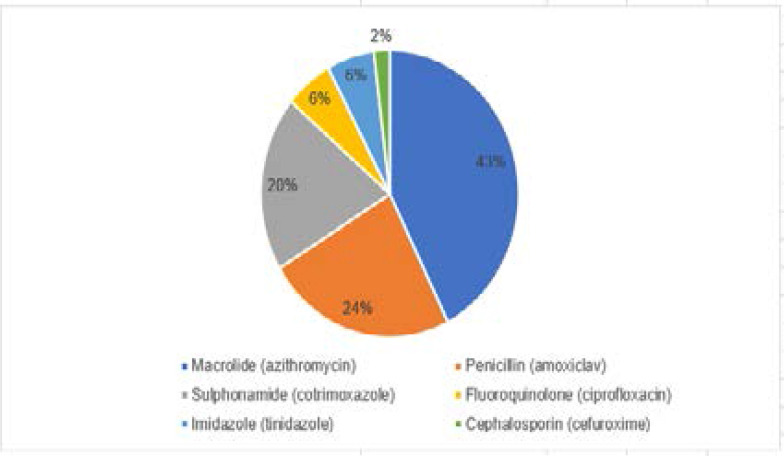
Antibiotics recommended for common cold

For common cold, 51/75 (68%) of the pharmacists recommended an antibiotic. Macrolide (azithromycin) was the frequently recommended antibiotic 22/51 (43%), followed by the penicillin (amoxicillin/clavulanic acid), 12/51 (24%). Figure 1 shows antibiotic recommendations for common cold.

**Figure 2:**
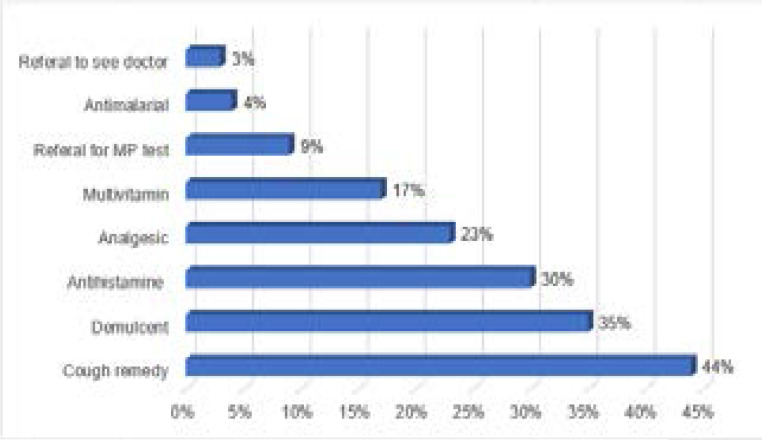
Other recommendations for common cold. MP – Malaria parasite

Among the pharmacists who recommended/dispensed antibiotics, 35/51 (95%) advised on how much of the antibiotics to take, 28/51 (76%) on how to take, and 20/51 (54%) on when to take. Only 13/51 (35%) gave a detailed description of how much to, how to and when to take the antibiotics. Majority (47/51, 92%) of the pharmacists who recommended antibiotic for common cold made other recommendations including, over-thecounter drugs and referral to doctor. Detail of other recommendations pharmacists made is shown in Figure 2.

### Acute (watery) diarrhoea

Community pharmacists tended to take more detailed history before recommending medications for acute diarrhoea, however, majority (did not ask if the patient was taking any other medicine(s). Summary of WWHWAM questions is provided in Table 3

**Table 3:** WWHWAM questions for acute diarrhoea

Questions	Yes, n (%)	No, n (%)
	N = 75	N =75
“Who is the medication for?”	66/75 (88)	7/75 (9.3)
“What are the symptoms?”	69/75 (92)	6/75 (8)
“How long have you had the symptoms?”	65/75 (86.7)	10/75 (13.3)
“What action has already been taken?”	37/75 (49.3)	38/75 (50.7)
“Are you taking any other medicine?”	15/75 (20)	60/75 (80)
“Other medical and lifestyle history?”	60/75 (80)	15/75 (20)

**Figure 3:**
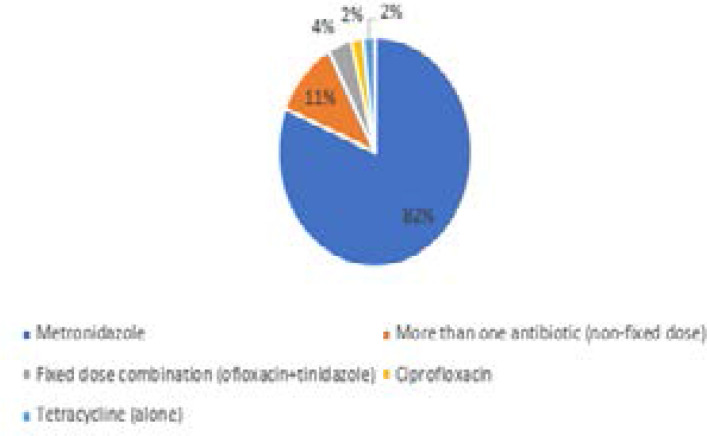
Antibiotics dispensed/sold for acute diarrhoea

For acute diarrhoea, 54/75 (72%) of community pharmacists recommended and/or dispensed an antibiotic. Among those who dispensed antibiotic, 8/54 (15%) dispensed more than one antibiotic. The most frequently dispensed agent was metronidazole 44/54 (82%), which was dispensed in addition to amoxicillin or tetracycline among pharmacists who dispensed more than one agent. Details of antibiotics dispensed for acute diarrhoea are shown in Figure 3.

**Figure 4:**
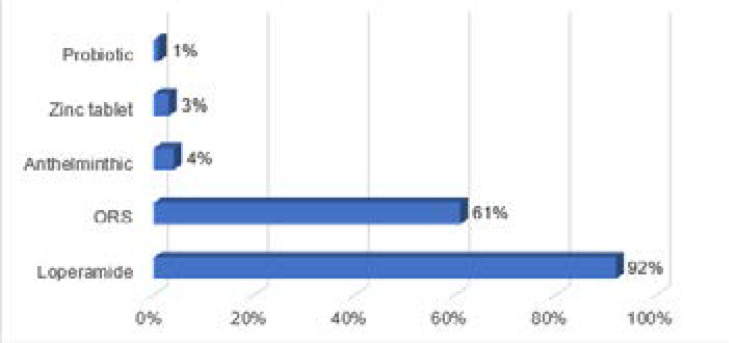
Over-the-counter recommendations for acute diarrhoea

Of the pharmacists who recommended at least one antibiotic, 47/54 (87%) provided advise on dispensed antibiotics, with 20/47 (43%) providing detailed advice on how much to, how to and when to take the antibiotics. Majority of the pharmacists, 72/75 (96%) dispensed OTCs recommended in treatment guidelines^26^,^27^ (example, loperamide, zinc) for acute diarrhoea, including those who dispensed antibiotics. Details of OTC recommendations are provided in Figure 4.

## Discussion

The objective of this study was to assess community pharmacists' management of common cold and acute diarrhoea, self-limiting infections that do not benefit from treatment with antibiotics. Findings of this study show high rate of inappropriate dispensing of antibiotics for self-limiting conditions. Overall, antibiotics were recommended in 68% of visits for common cold and 72% of visits for acute (watery) diarrhoea. There was only four percent difference between the proportion of antibiotics recommendation for acute diarrhoea and common cold; thus, antibiotics were misused at about the same level for both conditions. In this study, azithromycin and amoxicillin/clavulanic acid were the most frequently dispensed antibiotics for common cold, and metronidazole for acute diarrhoea. While these antibiotics have a wide range of indications, their inappropriate use in treatment of self-limiting infections results in decreased response to treatment by bacteria which were initially sensitive, and increased selection pressure for community antibiotic resistance. For example, in a study to compare macrolide resistance in communities treated with mass azithromycin for trachoma with untreated control communities, Skalet et al.^34^ reported a significant increase in azithromycin resistance to Streptococcus pneumoniae from 3.6% at baseline to 46.9% at the twelfth month of monitoring.

Previous studies^14^–^16^,^18^ on dispensing for self-limiting infections have shown that community pharmacists are willing to provide antibiotics for self-limiting conditions. In a study to assess antibiotic dispensing practices by community pharmacists, Horumpende et al.^35^ reported amoxycillin, ampicillin/cloxacillin, and amoxicillin/clavulanic acid were dispensed without prescriptions for cough in both part II and I pharmacies. The same authors reported dispensing of trimethoprim/sulphamethoxazole, azithromycin and erythromycin for fever and metronidazole for acute diarrhoea. Similarly, Abdelaziz et al.^17^ reported amoxicillin was dispensed in almost all of simulated patient visits; 97.6% and 99.1% for acute bronchitis and common cold, respectively with gaps in history taking and advice after dispensing antibiotic. Findings of the present study therefore confirm inappropriate dispensing practice among community pharmacists and highlights the need for interventions to improve practice.

Accurate and complete history taking is a core principle of pharmaceutical care, which can rule out similar diseases, detect potential drug-drug interaction, and prevent medicine related problems.^36^ In this study, history taking and case management varied among the participating pharmacies. Improved history taking (in acute diarrhoea scenario) did not necessarily result in better case management; 65/75 (87%) of the community pharmacists asked at least three history questions, while 7/75 (9%) asked more than three questions for acute diarrhoea. Both those who asked at least three and more than three history questions dispensed at least an antibiotic. For the common cold scenario, the proportion of antibiotics dispensed were equal for those who asked and those who did not ask history questions. Of the 75 pharmacists, fifty (67%) asked at least three history questions, out of which thirty-four (68%) dispensed at least an antibiotic. The provision of unnecessary medications adds extra financial burden to patients in real life cases. This is particularly a concern considering that a large proportion of the population in LMICs rely on the community pharmacists partly to save cost associated with hospital visits.^6^,^8^

Of note, 69/75 (92%) and 72/75 (96%) of the community pharmacists dispensed at least one OTC recommended in treatment guidelines^24^–^27^ for management of common cold and acute diarrhoea, respectively. This outcome indicates that community pharmacists know the proper case management but may be influenced by other factors to dispense antibiotics inappropriately. Factors which have been reported to influence community pharmacists' antibiotic dispensing include fear of patient's dissatisfaction and/or misconceptions about efficacy of antibiotics, need to ensure business survival, incomplete knowledge of patient's symptoms and weak medicines regulatory policies.^9^,^19^,^20^ These factors may have played a role in the dispensing pattern among community pharmacists in this study.

In light of the link between antibiotic use and development of resistance,^37^,^38^ findings of this study highlight the need for strengthening and/or formulation of national/state antibiotic policy which requires dispensing antibiotics only on prescription. Such policy should be followed by requisite enforcement to ensure compliance. The findings also call for raising awareness of the dangers of antibiotic misuse and resistance among community pharmacists. This can be achieved by making antimicrobial stewardship (AMS) a component of the mandatory continuing professional development (MCPD) programme organised by the Pharmacists Council. The Council can make completion of such course a prerequisite for license renewal.

With education and training in AMS, and participation in awareness campaigns such as, the WHO World Antibiotic Awareness Week, held in November each year to create awareness of antibiotic resistance,^39^ community pharmacists can implement strategies to reduce inappropriate antibiotic use. Although community pharmacy-based AMS is not well described,^40^ emerging literature suggests community pharmaist-led interventions, including collaborative practice agreements (CPAs), point-of-care testing and academic detailing are effective in promoting rational antibiotic use.^10^ While these strategies may not be applicable in all settings, especially in African countries with underdeveloped healthcare system, community pharmacists can reduce inappropriate antibiotic dispensing through adherence to available treatment guidelines (local or international) for commonly encountered infections. In this study, a small percentage of community pharmacists, 32% and 28% did not dispense antibiotic(s) for common cold and acute diarrhoea, respectively.

Furthermore, pharmacists are key members of AMS teams.^41^ To prepare pharmacy students for their future role in AMS in hospitals and the community, AMS need to be incorporated in undergraduate pharmacy programme. Available evidence indicates AMS is incorporated in undergraduate pharmacy curriculum in western countries.^42^,^43^ South Africa is currently developing an AMS curriculum to be incorporated in the South African undergraduate Pharmacy degree;^44^ Nigeria could join in this laudable feat and incorporate AMS principles in undergraduate pharmacy and other healthcare programmes.

The main limitation of this study is that it was a onestate study, during which we simulated only two self-limiting infections. Findings may not be generalised to the rest of the country and other infections. A nation-wide simulation study of other infections is needed to determine the scale of inappropriate dispensing among community pharmacists.

## Conclusion

This study shows a high rate of inappropriate dispensing of antibiotics in community pharmacies for common cold and acute diarrhoea, conditions which require no antibiotics. There is need for improved awareness of antibiotic resistance through awareness campaigns, continuing education and training of community pharmacists, and the inclusion of antibiotic resistance and stewardship in undergraduate pharmacy curriculum.
